# Spatial patterns of malaria in a land reform colonization project, Juruena municipality, Mato Grosso, Brazil

**DOI:** 10.1186/1475-2875-10-177

**Published:** 2011-06-26

**Authors:** Elaine Cristina de Oliveira, Emerson Soares dos Santos, Peter Zeilhofer, Reinaldo Souza-Santos, Marina Atanaka-Santos

**Affiliations:** 1 Epidemiological Surveillance, Health Secretary of Mato Grosso. Rua D, Political Administrative Center, Cuiabá, Mato Grosso State, 78.050-970, Brazil; 2 Department of Geography, School of Philosophy, Literature and Human Sciences, Av. Prof. Lineu Prestes, 338. Cidade Universitária. University of São Paulo, São Paulo, São Paulo State, 05.508-080. Brazil; 3 Department of Geography, Federal University of Mato Grosso, Av. F. Corrêa, Cuiabá, Mato Grosso State, 78.060-900, Brazil; 4 Department of Endemic Disease, Brazilian National School of Public Health, Oswaldo Cruz Foundation. Rua Leopoldo Bulhões, 1480, Rio de Janeiro, Rio de Janeiro State, 21.041-210, Brazil; 5 Institute of Public Health, Federal University of Mato Grosso, Av. Fernando Corrêa, Cuiabá, Mato Grosso State, 78.060-900, Brazil

## Abstract

**Background:**

In Brazil, 99% of malaria cases are concentrated in the Amazon, and malaria's spatial distribution is commonly associated with socio-environmental conditions on a fine landscape scale. In this study, the spatial patterns of malaria and its determinants in a rural settlement of the Brazilian agricultural reform programme called "Vale do Amanhecer" in the northern Mato Grosso state were analysed.

**Methods:**

In a fine-scaled, exploratory ecological study, geocoded notification forms corresponding to malaria cases from 2005 were compared with spectral indices, such as the Normalized Difference Vegetation Index (NDVI) and the third component of the Tasseled Cap Transformation (TC_3) and thematic layers, derived from the visual interpretation of multispectral TM-Landsat 5 imagery and the application of GIS distance operators.

**Results:**

Of a total of 336 malaria cases, 102 (30.36%) were caused by *Plasmodium falciparum *and 174 (51.79%) by *Plasmodium vivax*. Of all the cases, 37.6% (133 cases) were from residents of a unique road. In total, 276 cases were reported for the southern part of the settlement, where the population density is higher, with notification rates higher than 10 cases per household. The local landscape mostly consists of open areas (38.79 km²). Training forest occupied 27.34 km² and midsize vegetation 7.01 km². Most domiciles with more than five notified malaria cases were located near areas with high NDVI values. Most domiciles (41.78%) and malaria cases (44.94%) were concentrated in areas with intermediate values of the TC_3, a spectral index representing surface and vegetation humidity.

**Conclusions:**

Environmental factors and their alteration are associated with the occurrence and spatial distribution of malaria cases in rural settlements.

## Background

In Brazil, and more specifically in the state of Mato Grosso, the population in rural land reform settlements and occupations increased dramatically in the 1980s and 1990s. Additionally, thousands of people migrated to the northern regions of the state during this period, attracted by the discovery of gold there. Unregulated mining became a principal economic activity, increasing the occupation of the region [1] and leading to intense land use and dramatic environmental transformations in the Amazon landscapes [2].

The informal gold extraction techniques that predominate in these mining areas favor the creation of environments appropriate for malaria vector reproduction, with numerous bodies of standing water and small areas with high population densities [3].

As a consequence of the complex network of factors involved in the transmission of malaria, one technique for the analysis of the disease has utilized Geographical Information Systems (GIS). GIS allow information from different sources to be stored and integrated, enabling an analysis of the interactions between relevant variables in their spatio-temporal context, the development and testing of hypotheses, supervision and evaluation of interventions and the construction of predictive models as part of preventive operations [4-6].

As the spatial patterns of malaria in a territory reflect complex interactions between parasites, vectors, and human hosts, studies of the influences of environmental factors on malaria allow the simulation of epidemiological risk situations as a tool for health care decision-making and the definition of priority actions for malaria control [7]. Many large-scale studies exist, as shown by EISEN and WRIGHT [8], but studies at different spatial scales are required to understand fully the relationships between landscape features, parasite distribution, and malaria infection. As the risk of malaria infection is related to multiple factors, the interactions between which have rarely been explored at local scales in the southern Amazon, the present study identifies and analyses the influences of multiple socio-environmental characteristics on the spatial distribution of malaria cases in the "Vale do Amanhecer" settlement of the Brazilian land reform programme in northern Mato Grosso.

## Methods

### Study area

**Figure 1:**
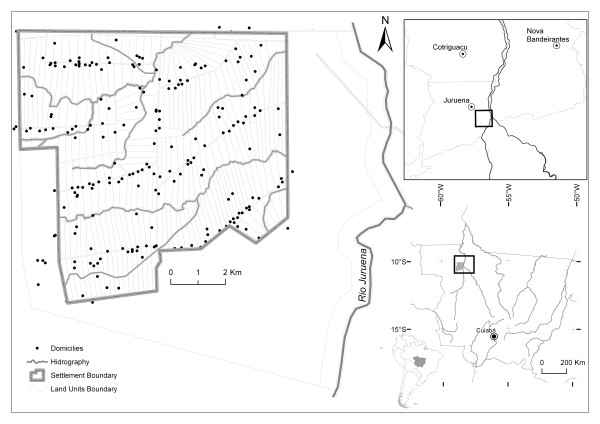
**Study area**.

The "Vale do Amanhecer" settlement is located in northern Mato Grosso, about 6.2 km from the city of Juruena, at 10°22'53.84" S latitude and 58°25'27.35" W longitude. Founded by the Federal Institute of Colonization and Land Reform [9] in 1998, the settlement comprises 14,400 ha. One half of the area was designated for settlement by 250 families, whereas the other was set aside as a permanent protection area. Each land unit has an area of about 26 ha, and units are distributed along projected, linear roads numbered from 01 to 14 (Figure 1). The average demographic density is 10.41 inhabitants/km^2^. The main economic activities of the settlement are cattle farming and agricultural subsistence and, mainly along the streams, some informal gold mining.

### Data acquisition and analysis

#### Epidemiological data

A total of 585 notification forms from the Epidemiologic Surveillance Information System [10] from the year 2005 were obtained from the municipal health care administration. All registries were cross-checked and georeferenced in a TerraView 3.1.4 [11] spatial database.

#### Spatial data layers

Spatial data sets were elaborated using TerraView 3.1.4 (INPE), ArcGIS 9.2 (ESRI), SPRING 4.3 (INPE) and ENVI 4.1 (RSI) software. A multispectral TM-Landsat 5 image from 25-06-2005 (WRS 229/67) was obtained from the Instituto Nacional de Pesquisas Espaciais (INPE) and georeferenced. A land-use map was created through the visual interpretation of a #3#4#5 color composite, where the following three thematic classes were differentiated: (a) Forests, (b) Agricultural area, and (c) Secondary vegetation.

Two spectral indices were derived from the Landsat TM imagery: the Normalized Difference Vegetation Index (NDVI), a widely used measure of the amount of green biomass, and the third component of the Tasseled Cap Transformation [12], an index representing the humidity of the first reflectance layer (vegetation cover or soil surface). The NDVI was calculated in the SPRING 4.3 software by the combination of the red (#3) and near infrared (#4) bands ([#3-#4]/[#3+#4]) scaled into 256 grey levels. Then, three classes were defined: low (0 to 100), intermediate (101 to 200) and high (> 200). The Tasseled Cap Transformation (hereafter TC_3) was performed by the respective module in ENVI 4.1 and then differentiated into the following classes: low (0 to 76.5), intermediate (76.6 to 165.8) and high (> 165.8).

The following distance layers were created using the buffer command of the TerraView 3.1.4 software: One for the distance to potential procreation habitats, with three 100-m zones (100, 200 and 300 m); one for the distance to streams, with 100-m zones between 100 m and 1,500 m; and one for the distance to mining areas, in 300-m zones from 300 m to 6,900 m. These values were selected considering knowledge of the feeding and reproduction habits of *Anopheles *species, which can fly further than 1.5 km but generally remain near their reproductive habitat [13].

This study is part of the "Malaria spatial analysis in rural land reform settlements project" and was approved by the Julio Muller Hospital Ethics Committee.

## Results

In 2005, the settlement was occupied by 718 inhabitants, 394 (54.87%) of them male and 324 (45.13%) female. A total of 359 malaria cases were reported, 23 of which were excluded from the sample because no residence address was given and 18 because the patient's residence was located outside of the settlement.

**Figure 2:**
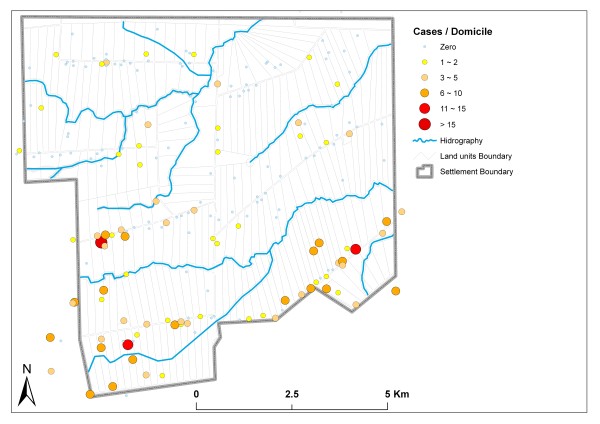
**Distribution of malaria cases**.

From the remaining total of 336 cases, 133 positive smears were from residents of Road 08, which corresponds to 37.60% of all reported cases. For Roads 13 and 05, 124 (35.10%) and 58 (16.40%) malaria cases were reported, respectively (Figure 2).

Cases were concentrated in the southern part of the settlement, where 276 cases were reported, often with more than 10 cases per domicile. In this area, 102 cases (30.36%) were caused by *Plasmodium falciparum *and 174 (51.79%) by *Plasmodium vivax*. The northern part of the settlement presented 60 cases, and most domiciles reported fewer than 10 cases per domicile. Of these cases, 16 (4.76%) were caused by *P. falciparum *and 44 (13.09%) by *P. vivax*.

**Figure 3:**
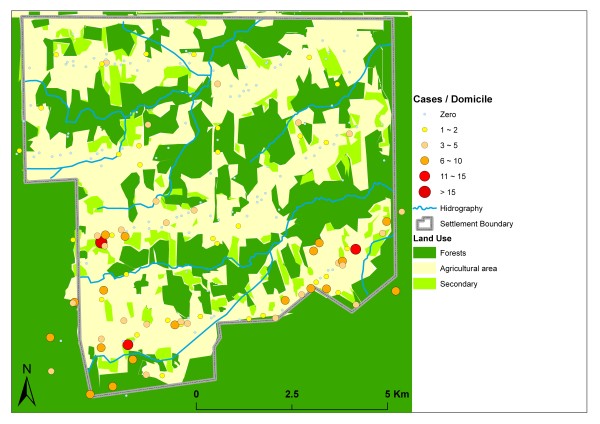
**Land use and malaria cases**.

The visual interpretation of the Landsat TM imagery showed that 38.79 km² of the settlement's land was deforested, including terrains used for housing, pastures and agriculture. Forest reminiscents occupied 27.34 km² and secondary vegetation 7.01 km² (Figure 3).

The concentration of malaria cases in the southern part of the settlement coincides with the predominance of deforested areas there and its proximity to most mining areas. As expected, 154 (75.86%) of the 203 domiciles (200 in the settlement proper and 3 in the permanent protection area) were located in open areas, which are generally characterized by low NDVI values (from 0 to 100). This result documents the intense land transformation process initiated with the occupation in 2000, which has included the transformation of natural vegetation into pastures, the opening of mines and the installation of mining equipment since 2002. Residents in this area presented 239 (71.13%) malaria cases, with an average of 1.56 per domicile (Table 1).

**Table 1:** Absolute (AF) and relative frequencies (RF), average (Av) and standard deviation (SD) of Land-use, NDVI and TC_3

Variables	Classes	AF	RF (%)	AF^1^	RF^1^	Av	SD	N. D^2^
**Land-use**	Agricultural area	239	73.31	239	73.31	1.49	2.84	160
	Secondary vegetation	17	5.21	256	78.53	2.13	4.85	8
	Forest	70	21.47	326	100.00	1.71	2.74	41
	**Total**	**326**	**100.00**	-	-	**1.56**	**2.90**	**209**

**NDVI**	Low	209	64.11	209	64.11	1.49	2.78	140
	Intermediate	64	19.63	273	83.74	1.28	2.96	50
	High	53	16.26	326	100.00	2.79	3.44	19
	**Total**	**326**	**100.00**	-	-	**1.56**	**2.90**	**209**

**TC_3**	Low	78	23.93	78	23.93	1.24	2.11	63
	Intermediate	151	46.32	229	70.25	1.70	3.44	89
	High	97	29.75	326	100.00	1.70	2.75	57
	**Total**	**326**	**100.00**	-	-	**1.56**	**2.90**	**209**

^1 ^- Accumulated

^2 ^- Number of domiciles

**Figure 4:**
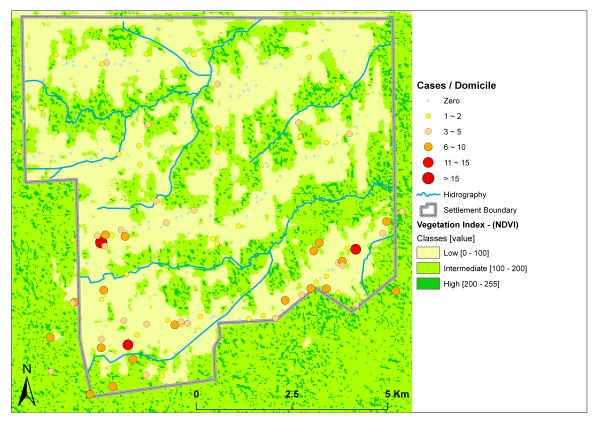
**Vegetation index (NDVI) and malaria cases**.

Whereas this area of the settlement, where the population density is highest, reported the majority of the malaria cases in terms of absolute numbers, the highest infection rates occurred in areas with denser vegetation. Domiciles located within or near the forested permanent protection areas with high NDVI values (> 200) reported 2.8 cases per domicile; the domiciles with more than five reported cases are located in areas of high NDVI values as well, mainly near the stream network (Figure 4).

**Figure 5:**
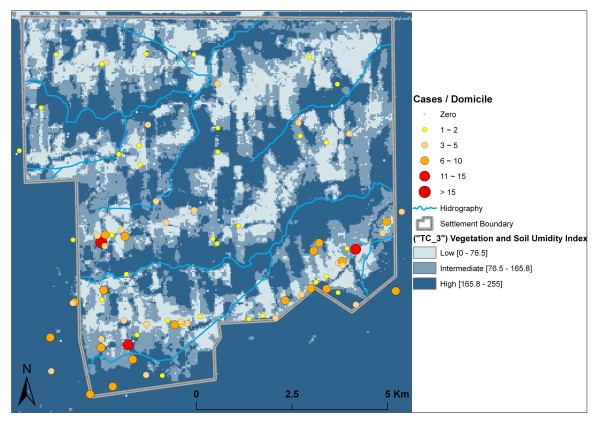
**Vegetation and soil humidity index (TC_3) and malaria case distribution**.

In absolute terms, the highest numbers of domiciles (41.78%) and malaria cases (44.94%) were located in areas with intermediate values of TC3. Humid surfaces with high values of TC_3 occurred mainly in densely vegetated areas and near the stream network. The average number of malaria cases per domicile in these areas (1.85) is slightly higher than that in areas with intermediate TC_3 values (1.82). The number of cases per domiciles is much lower (1.30 cases per domicile) in areas with low TC_3 values (Figure 5).

**Figure 6:**
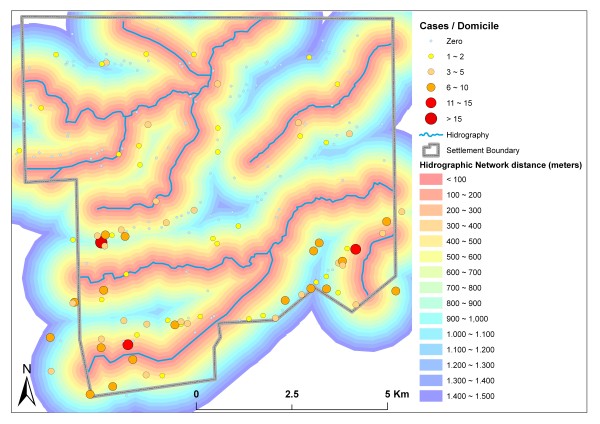
**Distance to nearest stream (*buffers*) and malaria cases**.

Domiciles reporting malaria cases were observed across the whole range of distances from the stream network. Most malaria cases, however, occurred in domiciles up to 900 m from streams. Domiciles with more than 15 cases were concentrated even closer to the hydrographic network, at a maximum distance of 300 metres (Figure 6).

**Figure 7:**
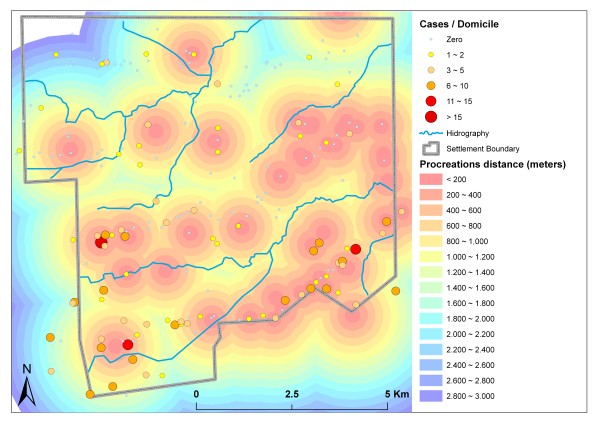
**Distance to nearest potential reproductive habitat (*buffers) *and malaria cases**.

All domiciles with malaria cases were located less than 1.6 km from the nearest potential reproductive habitat of Anopheles species, including watercourses, standing water, springs, wells and small dam reservoirs. Cases decreased on a gradient with increasing distance from these locations (Figure 7).

**Figure 8:**
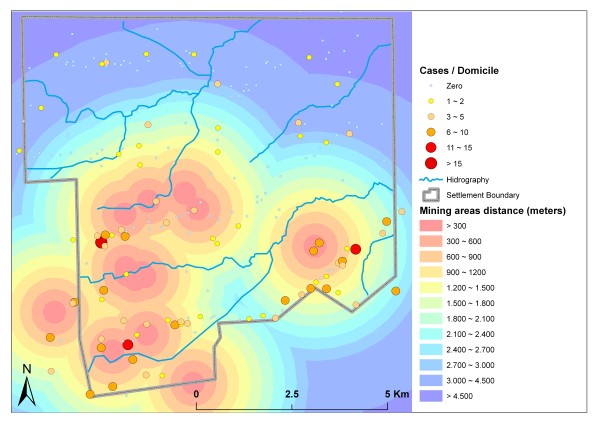
**Distance to nearest mining area and malaria cases**.

Cases were also negatively related to the distance from mining areas. The 70 domiciles (34.48%) located less than 1.2 km from a mining area accounted for 237 malaria cases (70.5%), with an average of 3.38 cases per domicile. All domiciles with more than five cases were located at most 2.1 km from the nearest mining area, whereas all domiciles with fewer cases (between one and five) were at least 1.2 km from the nearest mining area (Figure 8).

## Discussion

In the Vale do Amanhecer settlement, malaria was highly prevalent in 2005, with about 47% of the settlement's 718 inhabitants affected by the disease. Therefore, the study area provides a clear example of the focal nature of malaria occurrence in the Amazon region, where malaria is often concentrated in settlements and areas of mining activity. The average prevalence in the Juruena municipality is about five times lower than in Vale do Amanhecer.

The comparison of the spatial pattern of malaria occurrence with geographic layers representing vegetation distribution and distances to watercourses and mining areas showed variable findings in this fine-scale epidemiological study.

Numerous studies have shown that malaria infection is influenced by environmental factors, such as temperature, precipitation, humidity and altitude. In tropical regions, such as the Amazon region, year-round high temperatures and precipitation favor malaria transmission [14-16,2]. Most studies have been large-scale surveys on a regional or country scale [14,17,7]. As variations at these scales can be governed by other external factors, local-scale studies, such as those carried out within a single settlement [18], are crucial for understanding the driving factors of malaria transmission and for community-based health planning [19]. At this scale, transmission rates are not only governed by demographic or ecological factors but are also influenced by the social and cultural contexts of local populations [20].

In the studied settlement, relationships between the environment and spatial patterns of malaria occurrence were observed. For risk evaluation, the combination of land use and vegetation cover must be considered. More cases are reported in deforested areas with low NDVI and TC_3 values, but this is exclusively due to the higher local population density and population fluxes. The highest rates of malaria per domicile were reported within or near forest reminiscent with high spectral indices. Additionally, at a local scale, it must be considered that infection did not necessarily take place in the domicile.

Vegetation cover plays an important role in the biological cycles of vectors and infectious agents, particularly if other environmental conditions, such as precipitation, temperature or humidity, are altered [21,22]. This alteration explains the elevated incidence of malaria cases in domiciles inside or near forested areas with high values of NDVI, which may be characterized by higher vector densities. The low absolute numbers of cases in these areas are caused by their low housing density (18.2% of all domiciles) and low population flux.

High TC_3 values were observed inside forested areas and areas with dense vegetation succession. The highest average case rate per domicile (1.82) was found in areas with high TC_3 values, possibly due to the presence of humid areas favoring vector proliferation. These areas mainly coincide with buffer zones near streams, explaining the concentration of cases in domiciles less than 1 km from the hydrographic network, which is dense throughout the settlement.

High malaria infection rates in the settlement are supposedly related to the changes in land use carried out by a population that originated partially from non-endemic regions. FERREIRA [23] identified a malaria prevalence of 56.0% in individuals who migrated to the settlement from non-endemic regions, who have an infection probability 2.9 times higher than individuals from endemic regions, a difference supposedly caused by their low immunity and lack of knowledge about measures to protect against the disease [16,24].

Studies such as [7,21,25,26] have pointed out that the distribution and dynamics of malaria cases are related to the phase of land occupation. Deforestation, aggregated with an influx of migrants, frequently produces new reproduction and principal feeding habitats, favoring the occurrence of epidemics in communities with populations characterized by high mobility, low immunity and low risk perception [24,3].

Infection rates are also thought to be elevated due to the lack of an adequate sanitation infrastructure, which motivates inhabitants to construct their housing near streams to facilitate domestic tasks and personal hygiene. Vasconcelos *et al *[7] described a similar spatial pattern of domicile distribution in the Jacundá municipality, attributing elevated malaria infection rates to this lack of sanitation infrastructure.

The observed malaria infection rates can be explained by the great number of potential reproduction habitats and mining areas in the settlement; cases are highly concentrated near these areas, which favor the survival and circulation of the main vector, *Anopheles darlingi *[7]. Close relationships between mining activities and malaria cases in the Amazon region have also been reported by Santos *et al. *[27] and Duarte & Fontes [28].

## Conclusions

Configuration of land use with a mosaic of reminiscent forests and occupied deforested areas, a dense stream network, numerous breeding habitats and mining activity, paired with a mobile population with low immunity and low-risk perception, can explain the high malaria incidence in the studied settlement. Malaria, which is known to be a focal disease, presents a heterogeneous spatial distribution of cases, even in the current fine-scaled local study. Findings of this study demonstrate increased exposure to malaria in areas near potential mosquito vector breeding sites, namely near streams and mining areas and near more densely-vegetated areas.

## Competing interests

The authors declare that they have no competing interests.

## Authors' contributions

ECO and ESS contributed to the study design, data acquisition, processing and analysis and manuscript preparation. RSS, MAS, PZ, and ESS participated in the study conception, scientific coordination and revision of the manuscript. All authors read and approved the final manuscript.
